# Low Cost and Easy Validation Anticounterfeiting Plasmonic Tags Based on Thin Films of Metal and Dielectric

**DOI:** 10.3390/nano12081279

**Published:** 2022-04-09

**Authors:** Antonio Ferraro, Mauro Daniel Luigi Bruno, Giuseppe Papuzzo, Rosa Varchera, Agostino Forestiero, Maria Penolope De Santo, Roberto Caputo, Riccardo Cristofaro Barberi

**Affiliations:** 1 Physics Department, University of Calabria, 87036 Rende, Italy; bruno.mauro89@gmail.com (M.D.L.B.); maria.desanto@fis.unical.it (M.P.D.S.); 2 Consiglio Nazionale delle Ricerche-Istituto di Nanotecnologia (CNR-Nanotec), 87036 Rende, Italy; 3 Consiglio Nazionale delle Ricerche-Institute for High Performance and Networking (CNR-ICAR), 87036 Rende, Italy; giuseppe.papuzzo@icar.cnr.it; 4 Sicur Control System s.r.l., 87036 Rende, Italy; r.varchera@scs-web.it; 5 Institute of Fundamental and Frontier Sciences, University of Electronic Science and Technology of China, Chengdu 610054, China

**Keywords:** physical unclonable function, plasmonic, anticounterfeiting

## Abstract

Multilevel anticounterfeiting Physical Unclonable Function (PUF) tags based on thin film of silver (Ag), Zinc Oxide (ZnO) and PolyVinylPyrrolidone (PVP), are experimentally demonstrated and validated. We exploit the low adhesion of silver to glass and consequent degradation during ZnO deposition to induce morphological randomness. Several photographs of the tag surfaces have been collected with different illumination conditions and using two smartphones of diverse brand. The photos were analyzed using an image recognition algorithm revealing low common minutiae for different tags. Moreover, the optical response reveals peculiar spectra due to labels of plasmonic nature. The proposed systems can be easily fabricated on large areas and represent a cost-effective solution for practical protection of objects.

## 1. Introduction

In recent years, the number of counterfeit goods have been increasing at a fast pace within several sectors such as food, transport, data and cultural heritage to name a few. The illicit revenue is valued at hundreds of millions of dollars and is attracting more and more specialized workforce in this criminal activity. Apart from the huge financial losses for companies and people, this phenomenon represents a critical issue also for human health and safety [1]. To this end, the research community is strongly involved in developing new Physical Unclonable Functions (PUFs). The latter are authentication primitives largely used in cryptography applications that are now exploited to guarantee the authenticity of products, ensuring a high level of unclonability [2,3,4,5]. The main advantage of PUFs is related to the random and unpredictable arrangement of its constituents during the fabrication process which cannot be replicated even by the same manufacturer. The primitive responses are digitized and stored into a secure server and queried for authentication when necessary. The strength of each PUF depends on the number of challenge-response pairs (CRP) that it can support. For small numbers, it is categorized as “weak” while for large, as “strong” [6,7]. In this framework, chemical methods are widely explored for the realization of these systems because of the huge number of parameters that can be varied during the fabrication process [8]. Among them, diverse inks were formulated using lanthanides or other fluorescent molecules revealing information when excited by the proper wavelength [9,10,11,12]. Furthermore, during their application on a substrate, they produce luminescent random patterns increasing the overall anticounterfeiting performance [13,14,15]. A very interesting work has been recently reported in [16] where almost 10,000 tags have been realized using commercial printing and coating technologies. Some of them are validated using images acquired by smartphones equipped with a zoom lens. Morphological randomness can be also be produced by using dewetting, natural plants as template or sputtering technique [17,18,19]. In particular, the latter contribution illustrates three-level tags based on the percolation of few nanometers of silver thin film. Other approaches involving soft matter or disordered nanowires can be employed for the realization of microscopic fingerprints with high degree of uniqueness [20,21,22,23,24,24]. From a physical point of view, an advanced lithography technique as two photon direct laser writing can be considered for the realization of 3D nanometric anticounterfeiting labels [25,26]. The mentioned approaches, however, require expensive equipment and materials both for the fabrication and validation of the proposed tags. In view of inexpensive and easy end-user operation, we present an experimental study of weak PUF composed of silver, zinc oxide and polyvinylpyrrolidone deposited as a multilayer stack resembling a plasmonic optical nanocavity. The latter exhibits extraordinary properties as structural color depending on incident/view angle and characteristic transmission/reflection response [27,28]. Their validation is based on a low cost but very robust method. For the first security level, several photographs of random tag surfaces are acquired by smartphone and successively examined and validated by using an image recognition algorithm. This represents an overt protection level owing to the fact that it is a visible element. Since no complex apparatus it is necessary, this resembles a real life end-user operation. Instead, spectroscopic features represent a covert second security level requiring laboratory expertise. The presented multi level tags can pave the way for new anticounterfeiting systems, with inexpensive fabrication and operation, for the protection of consumer products as electronic devices, watchface, parfume bottle, car glasses, Murano vases et similia, or other goods where glass is present.

## 2. Materials and Methods

### 2.1. Tags Fabrication

The proposed anticounterfeiting tags are realized on 2 × 2 cm microscope glass slides by using a standard thin film fabrication technology. After cleaning the slides with Acetone and Isopropyl Alcohol, a Silver (Ag) layer of 30 nm is deposited using DC sputtering with the following parameters: vacuum 7 × $10^{-6}$, DC power 100 W for 60 s. Successively 80 nm of ZnO are deposited using the RF cathode at a power of 80 W and time of 26 min and 40 s. In total 9 labels have been created and from now on, they are referred with a number (#). The fabrication fo tags #1 to #5 is now complete. Successively, a solution of PVP in ethanol with a final concentration of 5 wt %, has been spin coated at 2000 rpm for 30 s followed by a baking on a hot plate at 80 °C for 5 min on tags from #6 to #9.

### 2.2. Image Recognition

All the acquired pictures have been analyzed through homemade image pattern recognition software that is able to identify unique features present into the image. This image recognition and feature-matching software application is based on the Scale Invariant Feature Transform (SIFT) algorithm [29]. The SIFT algorithm allows performing pattern recognition on 2D-images regardless acquisition angle, scale zooming and brightness changing. The execution phases of the algorithm are: (i) Constructing a Scale Space to ensure that the features are independents of the scale; (ii) Keypoint Localization to detect appropriate features (keypoints); (iii) Orientation Assignment to ensure rotation-invariant keypoints; and (iv) Keypoint Descriptor to describe each keypoint with unique fingerprint [30]. The detection of the suitable features (keypoints) consists in individuating positions and dimensions that can be assigned to the same object from different perspectives. The position can be detected by analyzing features with steady values for all possible dimensions, invariant to scale changes of the image. In order to detect a stable keypoint position in the scale space, a continuous scaling function, the scale space L(x,y,σ), is exploited. It is defined a L(x,y,σ)=G(x,y,σ)×Image(x,y), that is the convolution of a variable-scale Gaussian, G(x,y,σ), with an image, Image(x,y). The keypoints are characterized through a coherent orientation on the basis of the local image properties, that allows generating keypoint descriptors of this orientation and consequently invariant to image rotations. The gradient magnitude, m(x,y), and orientation, Δ(x,y) is computed through the pixel difference. For each feature point, the immediate neighbor in feature vectors of the current image, allows evaluating the conformity among feature points in the current image and the authentic image. The feature point with the lowest Euclidean distance for the invariant descriptor vector represents the immediate neighbor. A Java tool, built on open source software libraries (https://opencv.org/ (accessed on 4 January 2022), implementing the SIFT algorithm written in C++, was designed and implemented to analyze and compare the considered images.

### 2.3. Spectroscopic Characterization

For the spectroscopic analysis, a Halogen lamp (Ocean Optics DH2000, Orlando, FL, USA) is used as white source while a proper set of lenses is able to enlarge and collimate the beam diameter to 2 cm. On such a way, almost the entire tag area is investigated. The light is collected by an optical fiber connected to a UV–Vis spectrometer (Ocean Optics, USB2000+, Orlando, FL, USA).

## 3. Results and Discussion

**Figure 1:**
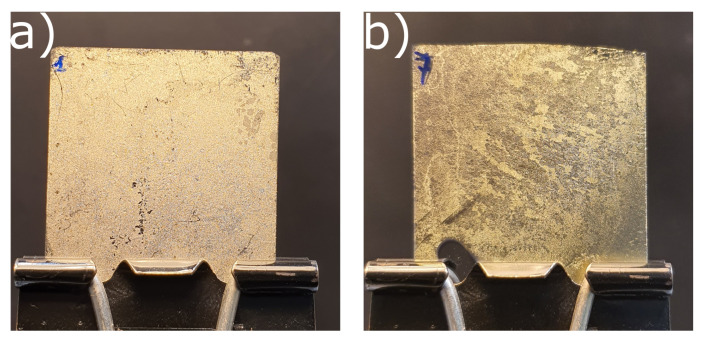
Smartphone Photographs of the fabricated tags composed of 30 nm of Silver (Ag) and 80 nm of Zinc Oxide (ZnO): (**a**) as deposited (Tag #1) and (**b**) covered with a 3 μm thick PVP layer (Tag #7). The pictures are taken with a black screen behind the Tags for collecting the reflected color.

The main anticounterfeiting feature of the proposed labels is represent by their morphological randomness which arise in the course of fabrication process. In fact, during the long deposition of ZnO, the Ag layer starts detaching from the glass surface with a random behavior. This effect derives from the low adhesion between glass and silver [31] together with the high deposition rate of Ag (30 nm/min) which does not give enough time for the Ag ions to be stabilized on the substrate. Figure 1a reports a photograph of the fabricated tag (Tag #1) acquired using a smartphone equipped with a photocamera with a 48 megapixel resolution. The image clearly evidences the randomness of the fabricated system which produces also a gold-like reflected structural color as designed using the approach reported in [28]. The color arises due to the fact that optical modes are confined at the Ag-ZnO interface [28,32,33,34]. As reported in Fabrication subsection, a PVP layer has been deposited on labels from #6 to #9 in order to increase the strength of the proposed tags. As observed in Figure 1b, which reports the photograph of tag #7, the use of solvent and temperature during the PVP deposition increases the randomness while preserving the structural color. For each fabricated tag, four photos have been acquired by using two different smartphones, equipped with photocamera having resolution of 48 megapixel and 12 megapixel, respectively, and illumination conditions. It is worth noting another important feature of the proposed tags, in fact, all photographs have been taken without any external lens attached to the smartphone thus enabling a low cost and easy implementation of the proposed anticounterfeiting system.

**Figure 2:**
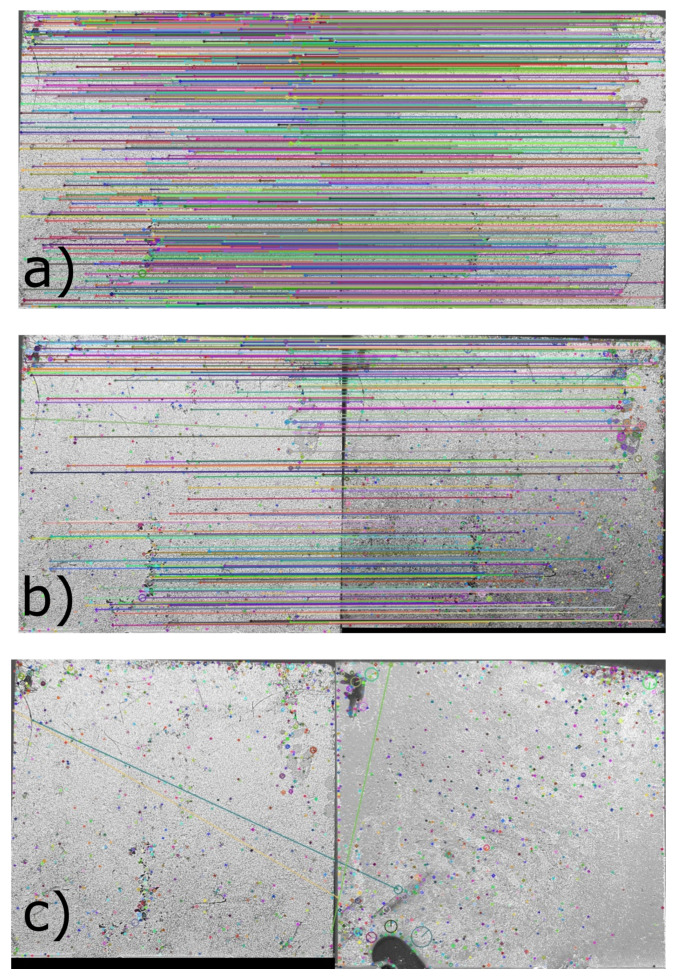
Tag Photo processing by recognition algorithm. Comparison between (**a**) the same photo of Tag #1 (**b**) two photos acquired with different smartphone of the same Tag #1 (**c**) photo of Tag #1 and Tag #7.

The acquired photographs have been analyzed using image recognition algorithm described in Section 2. Figure 2 reports an example of the recognized feature comparison between two images, where each common minutiae is linked with a color line. The more concurring points, the more robust the PUF. In particular, by analyzing a photo of Tag #1 with itself it is possible to obtain almost 900 common points as reported in Figure 2a. Instead, Figure 2b shows the comparison between two photos of Tag #1 acquired with smartphones of diverse brands. In this case, the common minutiae are almost 50. It is of paramount importance to say that the photos are taken with automatic settings and that each brand has its own algorithm to this purpose. However, this methodology corresponds to real life operation. It is worth mentioning that the required number of matching points in forensic fingerprint identification is usually in the few tens. Where the comparison is performed between different tags, a number of common features <5 is usually found as in the case illustrated in Figure 2c, which reports the result of tag #1 with respect to tag #7.

**Figure 3:**
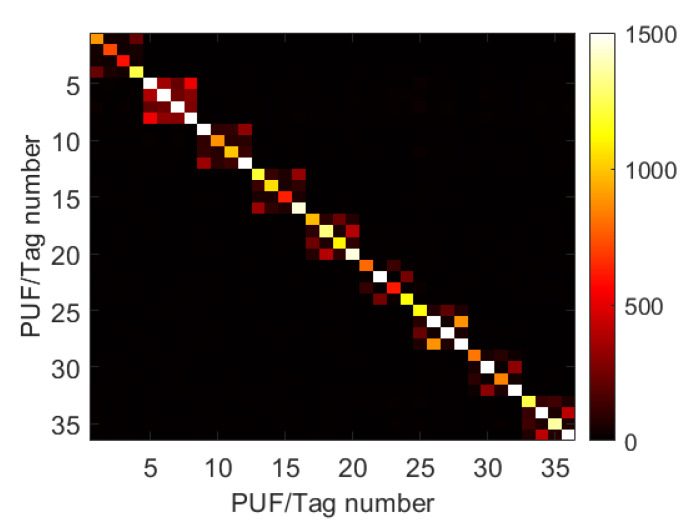
Correlation Maps of photos taken with two different smartphone and illumination condition of 9 fabricated tags.

The recognition/validation process was applied to all acquired pictures and the outcomes are illustrated in Figure 3, which reports the match scores map, namely the number of concurring minutiae between photos. A set of four different photos is available for each label. As expected the highest number of point lies on the diagonal meaning that different Tags have almost no common points [16]. Moreover, nine 4 × 4 matrices are clearly identified indicating that each tag can be validated also using different conditions with a very low-cost approach. In fact, photo of tags can be loaded into a certified database and, into daily life operation, the end-user just needs to take the photo and compare it with the registered one by using the developed app. The latter can also possess functionality for taking photos in the right way discarding, for example, the photos which are not focused.

**Figure 4:**
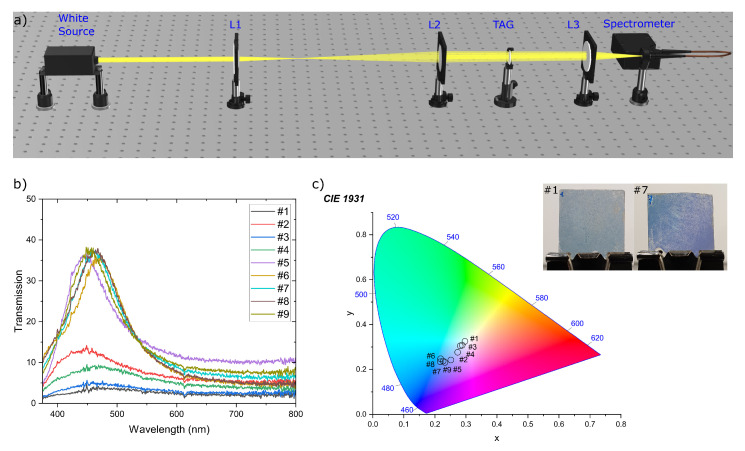
(**a**) Schematic drawing of the optical setup used for characterization; (**b**) transmission spectrum of 9 different tags; (**c**) CIE chromaticity xy-coordinate plots with the corresponding points. Inset: Smartphone Photograph of the fabricated Tag #1 (**left**) and Tag #7 (**right**). The photo are taken with a white screen behind the Tags for collecting the transmitted color.

It is important to underline that the illumination of the realized tags by white light excites plasmonic behaviors [28,35]. For this reason, the proposed PUFs manifest a second security level given by their peculiar transmission response. This can be used by specialized personnel into a second stage for confirming the results of the image recognition. In particular, a Halogen lamp (Ocean Optics DH2000) is used as white source while a proper set of lenses is able to enlarge and collimate the beam diameter to 2 cm. On such a way, almost the entire tag area is investigated. The light is collected by an optical fiber connected to a UV–Vis spectrometer (Ocean Optics, USB2000+)—schematic drawing of the whole setup is illustrated in Figure 4a. The acquired data is reported in Figure 4b showing a characteristic peak centered at a wavelength of about 450 nm for tags as deposited (#1 to #5). Instead, this peak is not present in the spectrum of tag covered by PVP (#6 to #9), even with a naked eye, no difference between the two tags can be observed. This behavior arises from the degradation of the multilayer structure due to solvent and temperature, and the difference is more evident by observing the CIE chromaticity xy-coordinates plots, calculated using an in-house developed Python script (Figure 4c). In fact, the PVP covered tags are identified by hues of white while the as deposited ones, by hues of blue. In the inset of Figure 4c, photos are reported of the tags #1 and #7 acquired using a white screen behind them in order to show the transmitted color. This is completely different from the reflected ones, passing from gold to blueish. The different chromatic behavior in reflected/transmitted colors, reported in Figure 1 and Figure 4, can be also exploited as a third level of authenticity. However, due to the fact that each smartphone camera has a different color and temperature settings influencing the final result, it is necessary to build a specific setup with a photocamera which will ensure reliability of photo taken at different times.

## 4. Conclusions

To conclude, we report on multilevel plasmonic anticounterfeiting tags realized on standard microscope glass slides by depositing silver, zinc oxide and polyvinylpyrrolidone. The unique morphology of each tag arises from the low adhesion of the Ag layer and the consequent degradation taking place during the ZnO deposition, thus leading to a very inexpensive label that can be identified as a weak PUF. The authentication and validation is ensured by simple image comparison, acquired by using common smartphones and analyzed by recognition software. A higher level of control, needed to guarantee the highest possible level of authenticity, is achieved by spectroscopic measurements. The presented tags constitute a low-cost solution for protection of high value goods in every day life.

## Data Availability

Data underlying the results presented in this paper are not publicly available at this time but may be obtained from the authors upon reasonable request.
